# The Implications on Future Ophthalmic Care During and Post-COVID-19

**DOI:** 10.3389/fpubh.2021.653708

**Published:** 2021-08-06

**Authors:** Qian Fan, Hongxia Wang, Wenjun Kong, Wei Zhang, Zhouyue Li, Yan Wang

**Affiliations:** ^1^Tianjin Key Lab of Ophthalmology and Visual Science, Tianjin Eye Hospital and Eye Institute, Nankai University Affiliated Eye Hospital, Clinical College of Ophthalmology Tianjin Medical University, Tianjin, China; ^2^Shanghai Guanghua Integrated Traditional Chinese and Western Medicine Hospital, Guanghua Hospital Affiliated to Shanghai University of Traditional Chinese Medicine, Shanghai, China; ^3^Beijing Youan Hospital, Capital Medical University, Beijing, China; ^4^State Key Laboratory of Ophthalmology, Zhongshan Ophthalmic Center, Sun Yat-sen University, Guangzhou, China

**Keywords:** COVID-19, ophthalmic care, optometry education, contact lens, mental health

## Introduction

COVID-19 has swept throughout the world and currently poses a global threat to public health (1, 2). After the whole world had entered a state of emergency (3, 4), strict restrictions were implemented on social interactions and travel, including domestic and foreign interactions. A large scale nationwide home quarantine brought about new ways of working and living. Robust public health responses and effective disease control strategies have been implemented in response to the COVID-19 pandemic. Such a massive mobilization to address an infectious disease is unprecedented. People are encountering massive difficulties in terms of prevention and effective control measures for this disease which does not have a consensus therapeutic protocol at the beginning. This global pandemic demands effective control measures and tremendous clinical practice pattern changes. However, there is still lack of big data concerning the mental health, contact lens (CLs) practice and optometry education. Therefore, it is necessary to reconsider these aspects for future ophthalmic care during and post the COVID-19 pandemic.

## Mental Health of the General Population and Frontline Medical Staff

The COVID-19 affects so many aspects of social life, many of which are related to being at risk for falling ill and straining the medical care system. Therefore, the public psychological states of the general population must not be overlooked (5). The COVID-19 pandemic may trigger a wide variety of psychological harm, such as anxiety, disappointment, depression, loneliness, isolation, fear, panic disorder, and other outcomes (6). The psychological condition of general population was valued through presenting questionnaire online during the pandemic. Non-anxiety and non-depression rates were 93.67 and 82.83%, respectively. There were anxiety in 6.33% and depression in 17.17% (5). Meanwhile, the mental and psychological status of medical staffs should also be stressed as important (7–9). Based on this, the State Council issued a new notice concerning the psychical and mental health of frontline medical staff jointly drawn up by several ministries including the National Health Commission (10). At the same time, an online screening inventory was also developed to help frontline medical staff find their possible mental problem and get timely psychological support through strategies to reduce psychological stress, the Psychic Hotline, and online diagnosis by professional psychiatrists (11). In addition, the use of real-time video connections, such as WeChat, Dingding, and Zoom etc., helped maintain important aspects of communication for frontline medical staff. In this crisis, a correct and comprehensive viewpoint toward disease is very important and indispensable. COVID-19 seems to be less deadly, although more contagious, than initially thought (12). This experience is a reminder not to neglect the mental health of the general population and frontline medical staff at the outbreak. At the same time, the adverse impact of COVID-19 on mental workload and mental health may also affect the social and emotional well-being of ophthalmic patients and ophthalmic healthcare workers. Precaution training, timely information communication, daily living supplies, and psychological counseling are indispensable. Everyone should be optimistic, positive, and confident toward overcoming this pandemic. Maintaining the good mental health is crucial for minimizing the adverse outcomes of this pandemic.

## Education for Ophthalmic Professionals

The COVID-19 pandemic maybe a double-edged sword, as it is not only a huge challenge to the public health but also a rare chance for the transformation of optometry education. Scholars have suggested that the increasing acceptance of such a transformation is due to its alignment with the biomedical models of professionalization, education, research, and practice (13). With the decline of the overall incidence rate of infectious diseases, the global trends of disease have gradually shifted to focus on chronic diseases such as cancer, cardio cerebrovascular diseases, and metabolic diseases (14). Even after severe acute respiratory syndrome (SARS), avian influenza, Ebola, and other infectious diseases, the global medical community was still too optimistic. The outbreak of COVID-19 posed a great threat to global health care systems. In general, the number of infectious department medical workers and related professionals is small, and the demand gap is large. Thus, when the outbreak of disease comes, sufficient talent reserves may be needed to ensure that the medical care system has an adequate response to the future waves of COVID-19 and does not enter into a prolonged depression that will further adversely affect the health of its people. This pandemic highlights the co-ordination not domination position of mental and psychological departments in many hospitals. In recent years, great achievements have been made regarding the public health. Under the present situation, the country is much more urgent to get the professional talents. The COVID-19 pandemic has also prompted us to strengthen the ability of optometry professionals to address major public health emergencies. Optometry education should add online training for infectious disease epidemiology, infectious disease control in high-risk settings, outbreak response and deployment to provincial and local public health departments to enhance the capacity to support a test, trace, track, and quarantine strategy (15). On the other hand, the pandemic may highlight optometrists talent shortage and urge the transformation of talent cultivation mode. Program graduates receive the Bachelor of Medicine degree (MD) from the universities but may assigned to work as optometrists by their employers since 2005 (16). However, a 2018 nationwide survey including 3,359 medical institutions, of which 1,463 hospitals had independent departments of ophthalmology at the county level, reported that full time optometrists and opticians were extremely scarce and urgently needed (17). The large shortage of eye care professionals urges to the cultivation of high-quality personnel in the future. The training mode of clinical optometrists is more mature in the medical education systems of developed countries. In the hospital eye services of United Kingdom (UK), optometrists undertake the traditional clinical roles of diagnostic refraction, color vision assessment, CLs fitting and dispensing, and low vision rehabilitation, as well as a wide range of extended clinical roles, which are traditionally performed by ophthalmologists (18). Optometrists in Singapore represent a skilled underutilized primary eye care provider by participating in screening and co-managing chronic eye conditions (19). Practice has suggested that composite optometry professionals with clinical medicine and public health backgrounds are often better qualified for the role of clinical experts, health policy decision-making, and leaders. Thus, the authors highlight the need for the new model of MD + Master of Public Health (MPH) or MD + continuing professional education (CPE) training, which should be studied and explored under national conditions in different areas. Although optometry professionals' level of primary eye care knowledge is high, their level of practical ability in screening and co-managing chronic eye conditions is low (19). Enabling their use for extended primary eye care roles would require further training. Exchange programs, conferences, and seminars further enhance educational standards by exchanging technical knowledge and good clinical practices. Regular CPE through e-learning is the most preferred mode, especially during the COVID-19 pandemic. The standardized training of optometry and optical practitioners, the strengthening of the prevention and control of infectious diseases and the improvement of the mental and psychological response abilities of optometry workforce are all needed. Comprehensive universities should seize the opportunity for a new round of scientific and technological revolutions. Strengthening the top-down design, improving the system and mechanisms, and building up excellent teaching staff are the keys to realizing the cultivation of talent. Coordinating medical schools, affiliated hospitals, public health colleges, pharmaceutical colleges, and other non-medical disciplines is inevitable in training optometry professionals. The COVID-19 pandemic is a rare chance to move forward regarding reforming the teaching model and incentive strategies. This pandemic has brought to us a new way of thinking about the training mode of optometry education, and will promote its reform and development.

## CLs Practice and Ophthalmic Surgery Practice in the Future

Furthermore, CLs practices have been changed by COVID-19, which is highly contagious with an extremely low incidence of ocular manifestations (20). Recent articles have reviewed the practices related to ophthalmic CLs wearers during the COVID-19 outbreak in America and European countries (21–23). The COVID-19 pandemic compromised the normal procedure of CLs wearers. A large number of CLs wearers chose to stay at home to avoid infection by experiencing population aggregation in optometric outpatient departments. To investigate the usage of CLs under this pandemic situation, the authors launched an anonymous online questionnaire through a professional platform which is named “Wenjuanxing” in WeChat software for mobile phone for data collection. This online questionnaire included 21 questions which focused mainly on the procedure change of CLs wearers. This online short-time survey included no personal information or ethical consent. A total of 289 CLs users were sent a two-dimensional barcode on WeChat to conduct this survey from April 20 to 27, 2020 in Tianjin, Beijing and Shanghai. Completed online surveys were collected from 132 CLs wearers for a good return rate of 46%. One hundred and twenty-one (92%) CLs wearers reported that they were living in a quarantine situation compared with the remainder (8%) who were working and living normally. Of the quarantine group, thirty (25%) reported using their CLs as usual, zero (0%) reported using their lenses more than usual and the remaining ninety-one users (75%) reported wearing their lenses less than normal. Of those working normally, six (55%) reported wearing their CLs as normal and five (45%) reported wearing their CLs less than normal. None of the respondents in this group reported an increased usage of CLs. The data of Chinese users were basically consistent with the European data (23). Most CLs users reported reducing their usage during COVID-19 pandemic. The reasons for the normal usage of CLs both in quarantine situations and working situations were the same as usual, including convenience, lack of burden, broader range of vision, better looking, and psychological comfort. In contrast, the reasons for the reduction in usage in the quarantine situation included fewer social opportunities, cost reasons, inconvenience in purchasing, more near-distance work opportunities, and less chance of infection. In normal situations, the lower usage of CLs was mainly reported as being due to daily precautions for virus; the protective spectacles were more favored. Infectious droplets and body fluids (such as tears and conjunctival secretions) can easily contaminate the human conjunctival epithelium; thus novel coronavirus may be easily transmitted through eye touching or rubbing (24, 25). Thus, the CLs practitioners should pay more attention to minimize the transmission of COVID-19 in CLs practice based on current scientific evidences (21). This survey was conducted quickly over the course of 1 week using people who had previously registered in three eye centers in Tianjin, Beijing and Shanghai. There are numerous limitations to this work. A larger sample population size should be included in the future to reduce the bias. More variables appropriate for the study should be taken into consideration, such as education background, age and sex distribution, city/location, length of wearing time, and type of CLs. The decreased need for CLs reflects the work and life pattern changes that have occurred during the COVID-19 outbreak. In the near future, CLs usage will increase as lives return to normal step-by-step. In addition, the COVID-19 pandemic has brought about clinical practice pattern changes, which should be viewed as an opportunity in a crisis. At the beginning of the pandemic, the Tianjin Eye Hospital implemented a variety of protocols to protect the staff and patients (26). These included establishing standard operating procedures according to the latest version of the Coronavirus Prevention Guidelines from China's National Health Commission and the US Centers for Disease Control and Prevention (CDC) guidelines and educating all employees on the procedures by hosting multiple drills. These measures included screening station to identify patients with fever; performing a temperature check and collecting epidemiologic information for every patient and visitor (up to one per patient) entering the hospital; setting up an isolation zone to screen patients in a well-ventilated area; avoiding or reducing close contact with patients; establishing a buffer zone and one-way movement in the hospital to reduce the risk of cross-infection between patients; reducing personnel in the hospital and advising them to limit their outdoor activities, and requiring reporting of health status by employees, as well as their cohabitant family members. All employees were asked to wear surgical masks, protective gowns, and goggles. Protective shields on devices such as asset lamps and non-contact chronometers were installed to reduce the risk of infection transmission from droplets. Frequent disinfection of the hospital, especially in the clinics and of medical devices were implemented everyday, and every clinic was equipped with air purifiers. Additionally, at the peak of the pandemic, the chronic disease and elective surgery wards were temporarily shut down, and only emergency services were provided (26). Telemedicine was also applied for patient consultations. Meanwhile, SARS-CoV-2 has the potential to be transmitted via aerosols posing a risk for ophthalmologists. The generation and distribution of aerosols from non-contact tonometry and the impact of tear film characteristics on aerosol generation were investigated by our team (26). One of the latest study from our team demonstrated that a 50 cm distance from the tonometer might confer safety from aerosols with <1.0-μm diameter. Patients with aqueous-deficient dry eyes would tend to generate more aerosols, and the use of protective eyewear in clinical settings for both doctors and patients were recommended (27). Good clinical protection can provide a feeling of safety for both ophthalmic care providers and patients. Personal protective equipment (PPE), such as protective suits and N95 respirators as well as masks, gloves and goggles, can be provided to reduce the psychosocial effects of fear and anxiety (28). Strict precaution measures should be persisted to protect both patients and practitioners in a long time. COVID-19 did impacted medical professionals in all fields of medicine and surgery in the academic, clinical, and surgical training. Ophthalmic surgery demands utmost accuracy and meticulousness, especially in corneal refractive surgery. It is imperative that wet-lab training should be included in the residency training program during the pandemic (29). Also, we have published a consensus in ophthalmic practice and surgery guideline (30).

## Discussion

COVID-19 has led to global changes in all trades and professions, which may be due to rethinking previous patterns. The COVID-19 pandemic has been a large test for the ophthalmic care system that includes mental health, professional education, and clinical practice. The implications of COVID-19 on future ophthalmic care may promote new styles of cooperative research on basic medicine, clinical medicine, and public health, which needs to be improved, especially cross-integration with non-medical disciplines such as the Internet, big data, artificial intelligence, and information science (31). In addition, cultivating the combined leading talents found in clinical optometry and public health represents the general trend. Furthermore, this pandemic reinvigorated patients' and doctors' attention to public health, the health needs, and health care services. When patients choose a health care provider, there may now be more focus on the facility and function, juxtaposed with higher demands placed on quality, safety, and professionalism. This will be the same demand for ophthalmic surgery patients and ophthalmologists. The pandemic could bring its service to a higher level, which can better improve patients' quality of life (30).

## Author Contributions

QF, HW, and WK designed and finished analysis, programming, and writing this article. WZ and ZL analyzed and gave comprehensive feedback on the manuscript. YW addressed all the feedback and finalized this manuscript. All authors have read and agreed to the published version of the manuscript.

## Conflict of Interest

The authors declare that the research was conducted in the absence of any commercial or financial relationships that could be construed as a potential conflict of interest.

## Publisher's Note

All claims expressed in this article are solely those of the authors and do not necessarily represent those of their affiliated organizations, or those of the publisher, the editors and the reviewers. Any product that may be evaluated in this article, or claim that may be made by its manufacturer, is not guaranteed or endorsed by the publisher.

## Abbreviations

COVID-19: coronavirus disease 2019
CLs: contact lens
SARS: severe acute respiratory syndrome
CPE: continuing professional education.
